# Bumpy ride ahead: Anticipated effort as emotional evidence?

**DOI:** 10.3758/s13415-024-01194-9

**Published:** 2024-05-14

**Authors:** Elad Oz-Cohen, Rotem Berkovich, Nachshon Meiran

**Affiliations:** Department of Psychology and Zelman Center of Neuroscience, Ben-Gurion University of the Negev, 84105 Beer-Sheva, Israel

**Keywords:** Anticipated effort, Feeling as evidence accumulation, Reaction time

## Abstract

Conscious reportable (un)pleasantness feelings were shown to be successfully described by a process in which evidence favoring pleasant and unpleasant feelings accumulates until one response wins the race. This approach is challenged by (a) insufficient specification of “evidence,” and (b) incomplete verification that participants report their truly experienced (un)pleasant feelings and not what they expect to feel. In each trial in this preregistered experiment, the (un)pleasant feeling reports regarding emotion evoking pictures was embedded in a period when participants expected a low-effort task (feature visual search) or a high-effort task (feature-conjunction search). Fitting the Linear Ballistic Accumulator model to the feeling report data shows that anticipated effort was associated with a higher rate of unpleasant evidence accumulation, but only when the emotion evoking pictures were normatively unpleasant and not when they were normatively pleasant. These results suggest that anticipated effort may be one source of “evidence,” but only given a certain interpretation of the findings, and that genuinely felt emotions contribute to the emotion reports, assuming that participants intended to react to the pictures, as instructed, and not to the anticipated effort.

**Supplementary Information:**

The online version contains supplementary material available at 10.3758/s13415-024-01194-9.

Imagine you are about to begin a new day at work. You enter your car and recall that you have extra work today. This mere anticipation of an upcoming effort causes your stomach to twitch, your skin to sweat, and your heart to beat faster. On your way to work, you notice how everything is perceived negatively: The news begins to sound alarming, and the passers on the streets seem angrier than usual. This small vignette describes the intuition that *anticipated* effort causes an unpleasant conscious experience. This intuition guided us in the present work, which concerns a framework and a model to describe consciously felt and reportable emotional experiences. Before describing the framework, model, and the specific goals of the work, we will provide a telegraphic review of the literature on (un)pleasantness feelings and anticipated effort.

## Anticipated effort and unpleasantness

The hypothesis that anticipated effort is aversive is corroborated in studies employing facial muscle recordings from the corrugator muscle (associated with frowning, and used as index of negative emotion) (Devine et al., 2023), skin conductance (Botvinick & Rosen, 2009), and patterns of brain activation (Klein et al., 2019). Perhaps most dramatically, Vogel et al. (2020) showed that when participants were given a choice, they were willing to trade upcoming cognitive effort for upcoming pain. However, these studies still do not address the question of whether anticipated effort is consciously experienced as aversive, because they did not measure the conscious and reportable experience itself. Specifically, according to the componential theory of emotion (Scherer & Moors, 2018), “emotion” should be described as a complex, multicomponent phenomenon consisting of aspects already studied, such as facial expression (Devine et al., 2023), physiological reactions (Botvinick & Rosen, 2009), and choice tendencies (Vogel et al., 2020). However, emotion additionally consists of feelings, the conscious experience, and how anticipated effort contributes to *emotional feelings* is yet to be explored. Dealing with this question proves to be a challenge, partly because emotional feelings are usually measured with *raw* self-reports. This is potentially a serious limitation, because raw self-reports are prone to numerous biases (Paulhus & Vazire, 2007). Several studies are informative with regard to our question despite the fact that their original goal was different than ours. Specifically, Dreisbach & Fischer’s (2012) work gets somewhat close to answering our question. These authors presented participants with congruent (easy) and incongruent (difficult) Stroop stimuli that served as primes and did not require a response. The primes were followed by affective pictures or words, and the participants’ task was to indicate whether this stimulus is positive or negative. The results show quicker responses to negative stimuli following incongruent (difficult) Stroop stimuli and quicker responses to positive stimuli following congruent (easy) Stroop primes. Dreisbach & Fischer’s results align with the hypothesis that anticipated effort causes an unpleasant feeling. Nonetheless, their task still does not fully match our needs because of two main reasons: (1) the Stroop stimuli that served as primes just reminded of effort and did not predict actual future effort. This is because there was no need to perform the Stroop task at that stage. In other words, the manipulation did not clearly involve anticipated effort; and (2) participants were asked to describe *the pictures/words* as negative/positive and not to describe *how they felt* when watching these stimuli. Namely, what was measured was *not* emotional experience, but rather, knowledge about it. We will return to the latter issue below.

In contrast to Dreisbach & Fischer’s (2012) results that support the hypothesis that anticipated effort causes unpleasant feelings, whether effort is aversive is nonetheless debated (David et al., 2022). As just one example, a recent work shows that participants often prefer performing a more difficult task over not doing anything (Wu et al., 2023), a fact that at least suggests that effort *investment* is not always aversive. As it turns out, a key issue is whether the effort is *expected*, as opposed to being *actually invested*. Specifically, Schouppe et al.’s (2015) results resemble Dreisbach & Fischer’s (2012) basic finding, but these authors additionally showed a reversed trend (greater liking for incongruent stimuli) after participants have reacted successfully to the Stroop stimuli (Bogdanov et al., 2022). These authors discussed their results in terms of Silvetti et al.’s (2011) computational model, according to which *anticipated effort* is associated with an increase in predicted failure (and is thus aversive). However, subsequent success in the (difficult) task increases the pleasant experience, because this success is relatively surprising and unpredictable. This approach suggests that whether *spent effort* is aversive may be questionable (although see David et al.’s, 2022, meta analytic results suggesting it is), but there seems to be a relatively broad agreement that *anticipated* effort *is* aversive. This broad agreement helped us deal with several challenges in the framework describing the emergence of conscious reportable (un)pleasant feelings as evidence accumulation.

## Emotional feelings as evidence accumulation

In a recent series of works (Berkovich & Meiran, 2023; Givon et al., 2020, 2023; Singer-Landau & Meiran, 2021), our lab used evidence accumulation modeling to describe the emergence of conscious reportable emotional feelings. Our paradigm involves asking participants to report, using a key press, if a stimulus makes them feel pleasant versus unpleasant. We then fit a mathematical model of speeded perceptual decisions to these data comprising the reports and associated reaction times (RTs). The intuition is that reporting about (un)pleasant experiences entails a choice (whether to report that I feel pleasant or unpleasant) and that this choice closely resembles perceptual choices. This assumption is borrowed from the perceptual theory of emotion originally proposed by James (1884) and later expanded by Laird & Lacasse (2014), among others. The theory posits that conscious emotional feelings resemble regular perceptual sensations. Adopting this assumption opens the way to employ highly developed models borrowed from perception theory to the study of emotional feelings. These include Signal Detection Theory (Macmillan & Creelman, 2004), which has been applied to emotional feeling reports (Karmon-Presser et al., 2018) and Evidence Accumulation Models (Forstmann et al., 2016) like the Linear Ballistic Accumulator model (LBA, Brown & Heathcote, 2008). The LBA is most relevant for the present work and is described in greater detail below.

## The linear ballistic accumulator model

The Linear Ballistic Accumulator (LBA) was originally developed to describe the cognitive processing taking place when making speeded perceptual decisions. An example for a two-choice perceptual decision is deciding whether a stimulus comprising red and green dots has more green dots (Response A) or more red dots (Response B). In our works, we used the LBA to explain how participants decide whether a stimulus makes them feel pleasant or unpleasant.

In describing RT, the LBA differentiates between the time taken by the decision itself (*decision time*) and the time taken by *nondecision* processes, including the early registration of visual features that takes place before any decision can be made and the time to prepare a motor response (such as a key press) after the decision has been reached. Critically for the present investigation, the decision itself is described by a race between evidence accumulators, with each accumulator gathering evidence favoring a given decision. For example, when reporting whether a picture feels pleasant or unpleasant, there are two accumulators: One gathering evidence favoring a “pleasant” response and the other accumulator gathering evidence favoring an “unpleasant” response. Two key parameters describe the decision process: The (across trials) average rate of evidence accumulation, the *drift-rate, mean_v*, and the amount of evidence required in order to reach a decision (*boundary, B*). According to the LBA, once the amount of evidence in an accumulator has reached boundary, this accumulator takes control over the response. For example, if the amount of evidence in the “pleasant” accumulator reaches boundary first, the response will be “pleasant.”

**Fig. 1:**
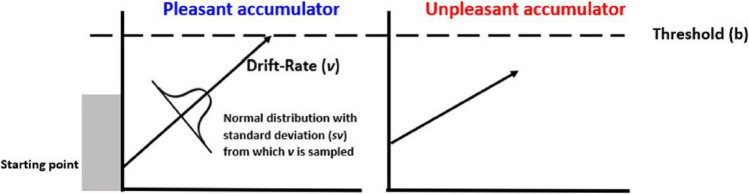
Hypothetical trial in which the participant reported experiencing a "pleasant" feeling. The horizontal axis represents time; the vertical axis represents evidence. The “pleasant” response was executed, because its accumulator was the first to cross the boundary. The slope of the arrow in each accumulator represents its mean evidence accumulation rate (drift-rate). This speed changes on a trial-by-trial basis such that in each trial, the value is sampled from a normal distribution with mean = drift-rate and a standard deviation ‘s_v.’ This figure was adapted with permission (from Berkovich & Meiran, 2023)

While the drift-rate describes the clarity by which the stimulus drives an (un)pleasant feeling, the boundary describes participant’s tendency to respond quickly despite not being fully certain versus respond later, once a high degree of certainty in the decision has been reached. A higher boundary suggests a more cautious approach, requiring more information before deciding. Conversely, a lower boundary indicates quicker decision making, potentially with less information Fig. 1.

Importantly, the LBA can be fit to empirical results (the choices and the entire RT distribution of each choice) to estimate the values of its parameters in a given context, in a manner analogous to how multiple regression and similar models are fit to data. The estimates, including drift-rate and boundary) can then serve as new dependent variables describing the emergence of (un)pleasantness reports.

The studies show an excellent fit of the LBA to emotional feeling reports. This has been demonstrated with visual fits by Givon et al. (2020) and then using the Root Mean Square Error of Approximation, RMSEA index (Schubert et al., 2017). Specifically, while the benchmark value of acceptable fit is RMSEA ≤ .08, the fits in our studies (Berkovich & Meiran, 2023; Givon et al., 2023) are one order of magnitude less (better) and in the same ballpark as a benchmark “standard” perceptual decision task.

Another key component in the process of model validation is demonstrating *selective influence*, indicating that external variables are each selectively and meaningfully associated with a specific LBA parameter. Accordingly, Givon et al. (2020) showed that slightly more intense stimuli were selectively associated with a higher drift-rate. Later works by Berkovich & Meiran (2023) shown this relation to hold throughout the valence range. Specifically, they studied the 2 to 8 range on a scale on which the norms were gathered, ranging from 1 (extremely unpleasant) to 9 (extremely pleasant). Berkovich & Meiran have actually demonstrated the drift-rate as a ratio scale for (a proxy of) emotion intensity, with a true zero and a constant unit. This enabled them to further show that emotional feelings, thus measured, follow Weber’s Law, which is the most basic law of sensation. Additional works show a selective influence of cognitive appraisals on the drift-rate (Singer-Landau & Meiran, 2021) and selective influence of speed-accuracy emphasis on the boundary (Givon et al., 2022).

An important advantage of this paradigm in the present context concerns the fact that our hypothesis regards changes in the *intensity* of the felt emotion. For example, we stipulate that unpleasant emotion would become even more unpleasant when anticipating effort. The fact that participants made a dichotomous pleasant-vs.-unpleasant response and did not rate the degree of (un)pleasantness made the current measure of degree (drift-rate) an indirect measure. As such, it was probably relatively less prone to demand characteristics and similar biases compared with direct intensity ratings.

## Challenges tackled in this work

In addition to addressing the core question (Is anticipated effort consciously experienced as unpleasant?), the present work addressed two other challenges that are specifically related to the feeling as evidence accumulation framework.

### What is “evidence”?

A central term in the model is “evidence,” and we wished to better characterize it. Karmon-Presser et al. (2018) and Givon et al. (2020) speculated that the potential sources of emotional evidence are something akin to the components of emotion (Scherer & Moors, 2018). These include the sensation of autonomic reactions, bodily expressions, cognitions, and action tendencies. Yet, these statements remain as speculations until empirical support is provided. So far, only one study characterized “evidence” (as involving cognitive appraisals) (Singer-Landau & Meiran, 2021). Here, we wished to broaden this investigation and explore anticipated effort as yet another possible source of emotional evidence.

### Do participants report genuinely felt experiences?

The other challenge concerns whether participants report genuinely felt emotions. For the LBA parameters to describe the emergence of (un)pleasantness reportable feeling, one must *assume* that participants report what they truly feel as opposed to what they think they are expected to feel. Whether this is true remains an outstanding question. We had so far addressed the issue by comparing semantic feelings to affective feelings (Itkes et al., 2017). Specifically, according to Itkes, Kron, and their associates, semantic feelings reflect an expectancy. This expectancy is based on previously acquired knowledge regarding how other people or I would feel when presented with a given stimulus. In contrast, affective feelings reflect the current experience. The distinction highlights the fact that what a person expects herself or others to experience may be different from what she currently experiences. Itkes, Kron, and their associates have accordingly shown several empirical dissociations between semantic and affective valence (Hamzani et al., 2020; Itkes et al., 2017; Itkes & Kron, 2019).

In one experiment in Givon et al. (2020), two groups of participants received detailed instructions to report either semantic feelings or affective feelings. For example, in the semantic condition, participants were asked to report “pleasant” (for example) if they knew that most people would regard the stimulus as pleasant even if they currently did not experience any pleasant feelings. In the affective condition, the instructions were flipped: respond (e.g.) “pleasant” if you feel a pleasant feeling even when knowing that most people would regard this stimulus as unpleasant or when you expect yourself to feel unpleasant. The results confirmed that the manipulation has worked, showing a higher rate of normative responses in the semantic condition as one would expect. More importantly, the LBA modeling results showed a significant difference between reports of sematic feelings and of affective feelings in the parameters describing the underlying process. This finding indicates that the process giving rise to reports concerning semantic feelings is (parametrically) different from the process that gives rise to reports concerning affective feelings.

Showing processing differences (Givon et al., 2020) still falls short of showing that reports of affective feelings are indeed about affective feelings. In the present work, we wished to take this issue one step further by showing that the drift-rate is selectively influenced by an implicit manipulation (predicted effort) that is known to generate unpleasant feelings.

## The current experiment

To address our goals, we designed a variant of the affective priming task where each trial consisted of a cue, announcing the type (and difficulty) of an upcoming visual search task (henceforth, VS-cue). The VS-cue was immediately followed by an affective picture requiring participants to reports, using a key press, about the (un)pleasant feelings that the picture has brought up. The (un)pleasantness reports concerning the picture were immediately followed by the visual search task. In other words, the (un)pleasantness judgment task was embedded in a period in which participants expected an upcoming task that would be either easy or difficult.

To manipulate anticipated effort, we varied the difficulty of the visual search task based on classic theory and findings by Treisman & Gelade (1980). The task required participants to detect a blue square among distractors. In the easy condition, the distractors and the target differed with respect to a single feature. They were either all yellow squares (color search, because what differentiated the blue square target from the yellow square distractors was color) or all of them were blue circles (shape search, because what differentiated the blue square target from the blue circle distractors was their shape). In the difficult condition, the target was surrounded by both blue circles and yellow squares intermixed, forcing participants to jointly consider both color and shape. As correctly pointed out by one reviewer, our manipulation involved a narrower construct than “effort,” because it pertained to cognitive load, which is just one type of effort.

Importantly, in this paradigm, not only the degree of unpleasantness but also the link between the expected effort and the (un)pleasantness report was implicit, like in Driesbach & Fischer (2012). This is because the (un)pleasantness report concerned the emotion evoking *picture* and not the *predicted effort*. We reasoned that if predicted effort would change the drift-rate, the change would minimally involve semantic feelings. Thus, such a finding would help showing that the task, and the estimated drift-rate reflect affective valence, at least to some extent, and would additionally show that anticipated effort consists a source of “evidence” in the evidence accumulation process.

Although the primary data concerned participants’ (un)pleasantness reports, which thus reflect emotional feelings in the usual sense, the parameters that we derived helped overcoming yet another challenge associated with report bias. Specifically, given that the LBA separately quantifies boundary and drift-rate means that (as long as the model is reasonably correct), the estimated intensity (drift-rate) if free of report tendency (boundary).

The analyses reported include a preliminary stage in which we fitted the LBA to the feeling reports data. This stage generated LBA parameter estimates, which served as the primary dependent variables in the core Bayesian Analyses of Variance (BANOVAs). Of interest are two parameters. The first is mean_v.normative, which is the drift-rate of the accumulator representing the normative report to the given picture, e.g., if the norm is below 5 the normative response is “unpleasant.” The second is mean_v.aberrant, the drift-rate of the accumulator representing the counter-normative report.

Our predictions (aside from showing good fit of the LBA, which we regard as a prerequisite) concern the BANOVA results:

- H1: trials with normatively unpleasant emotional stimuli that are coupled with a difficult (compared with an easy) visual search would have a higher mean_v.normative and a lower mean_v.aberrant.
- H2: trials with a normatively pleasant emotional picture that are coupled with an easy (compared with a difficult) visual search would have a higher mean_v.normative and a lower mean_v.aberrant.

The logic behind these two predictions goes as follows: If anticipated effort adds emotional evidence (easy = pleasant, difficult = unpleasant) then a cue indicating such effort would add emotional evidence favoring the corresponding (un)pleasant response.

## Method

### Participants

Seventy Israeli students (53 females, mean age = 23.5, range 21–33) from Ben-Gurion University of the Negev participated in this study in exchange for course credit. Before the experiment, all participants signed informed consent. All participants declared having fluent Hebrew (the language spoken at the University and the one used in the experiment) and normal (or corrected to normal) eyesight. Given that we are unaware of a proper sample size calculation, we used an approximation using G-Power (Faul et al., 2009). This calculation is clearly suboptimal, because it was done for Null Hypothesis testing (NHT) repeated measures ANOVA and not for the Bayesian analyses (BANOVA) that we employed. In our rough estimation, Alpha and Beta were set at .005 and .995, respectively. The resultant sample size was determined to be 73, but given that this is just an approximation, we ran only 70 participants.

### Design

The experiment involved two within-subjects independent variables: picture valance (positive or negative) and visual-search difficulty (easy vs. difficult). Both variables were fully mixed within a block.

### Apparatus

Because of the COVID-19 crisis, the experiment was run using the online web-based version of OpenSesame (Mathôt et al., 2012), hosted on a JATOS server (Lange et al., 2015), while the participants were at their homes.

### Stimuli

#### Cues for the search task

There were three possible cues, all indicating the type of distractors (and thus the type of visual search). The first cue indicated an upcoming color search (easy). This cue consisted of two yellow squares sized 0.5 x 0.5 cm, assuming a 17” monitor. The second cue indicated the shape search (easy). This cue consisted of two blue circles with a diameter of approximately 0.4 cm (assuming a 17” monitor). The last cue indicated conjunction search (difficult). This cue consisted of a yellow square and a blue circle of the same size as before.

#### Emotion Task

A total of 240 emotion-eliciting stimuli (half negative and half positive) with established norms (NAPS, Marchewka et al., 2014) coming from four different content categories (faces, objects, landscapes, and people) served as stimuli. Stimulus valence for the negative pictures was 2–4, whereas stimulus valence for the positive picture was 5.5–6.5, on a scale from 1 (very unpleasant) to 9 (very pleasant). The emotional stimuli were assigned into four similar content sets (sets 1–4). In hindsight, we realized that the pleasant and unpleasant pictures were not equated in terms of their distance from the neutral point (5), and we thus reran the core analyses on a subset of distance-equated stimuli. This non-preregistered analysis is reported in the Supplementary Materials.

In choosing the stimuli, we regarded only valence and not arousal. Our choice is based on results taken from similar stimulus sets, showing that arousal increases nearly linearly with the distance from the neutral point (Kron et al., 2013).

#### Visual Search Task

In this task, a total of 240 stimuli were used. These stimuli were generated using a custom code written in MATLAB R2021a. Within the shape search task (easy), half of its trials were target trials consisting of 1 blue square among 20 blue circles, whereas in the nontarget trials, all 21 shapes were blue circles. Within the color search task (easy), target trials consisted of 1 blue square among 20 yellow squares while nontarget trials consisted of 21 yellow squares. Finally, the target trials in the conjunction task (difficult) consisted of 1 blue square among 10 blue circles and 10 yellow squares, whereas in nontarget trials, there were 11 blue circles and 10 yellow squares. In all three tasks, all shapes within each stimulus were created such that (a) the minimal/maximal gap was set to be 24/300 pixels, respectively, and (b) the location of both the distractors and target was chosen at random. Finally, the size of the blue/yellow squares was 1 x 1 and the blue circles were 0.4 size in diameter.

### Procedure

**Fig. 2:**
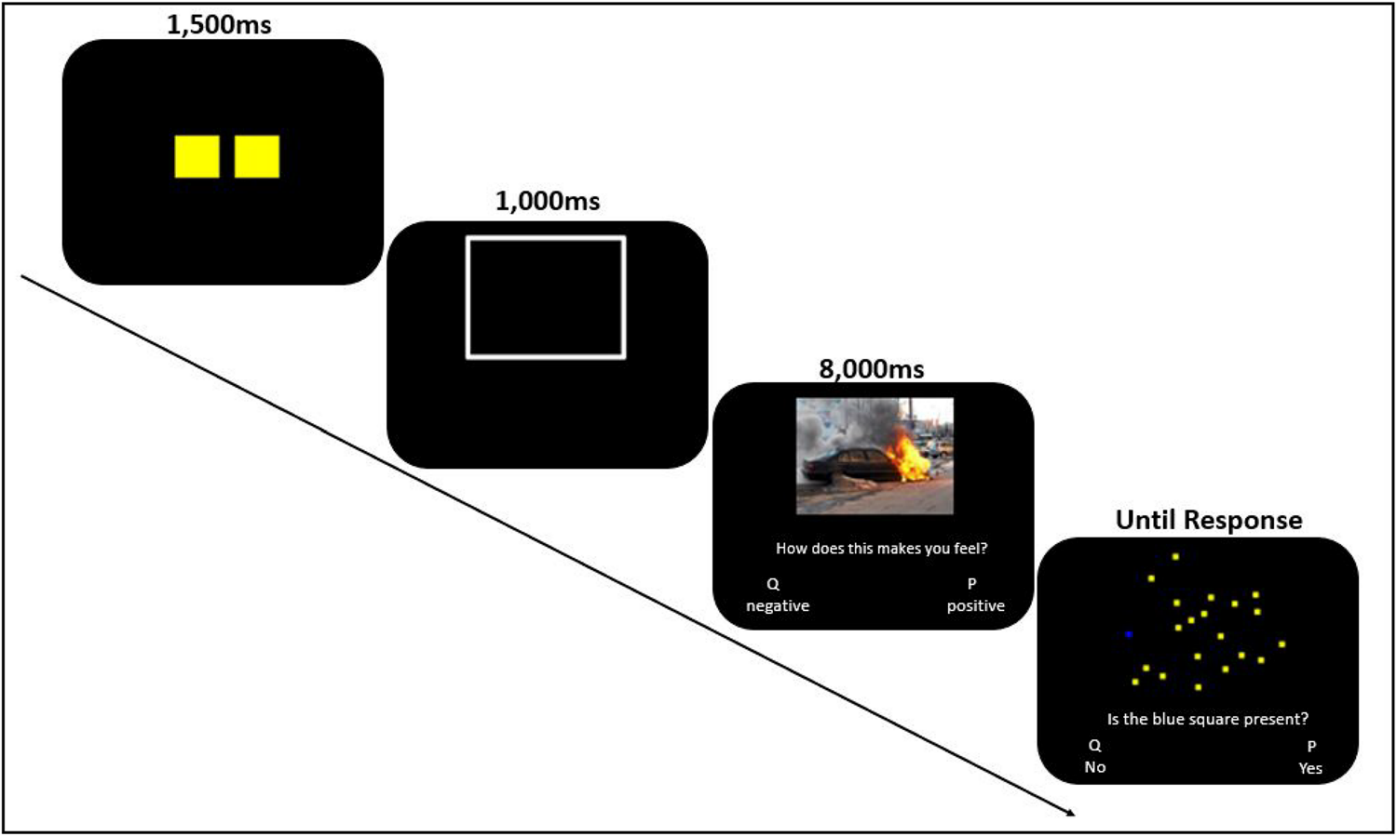
Experimental trial in the easy visual-search condition. The text was originally displayed in Hebrew—the language spoken at the University

Figure 2 depicts the general procedure. After the participant signed up for a particular time slot, the experimenter sent him/her a link to an online consent form to be completed before the signed time slot. All participants completed an informed consent before their assigned time slots. After completing the form, the experimenter sent a link to a video conference at the time slot to which they registered.

In the video conference, the experimenter explained that the goal of the study was to test the possible influence of emotion on visual search abilities. Consequently, in each trial, s/he will be presented with an emotional evoking stimulus followed by a visual search task serving as a metric for their visual search ability. Describing the goal of the experiment in this manner was deliberately false and served to reduce the influence of demand characteristics in the emotion reports. To further emphasize the visual search task as the target task, this task was described first. The description of the emotion report task was similar to that in the affective valence condition in Givon et al. (2020), emphasizing the subjective nature of emotion, the fact that we are not interested to know about the expected emotion, but in what the participant genuinely felt even if this feeling is not what most people would feel in that situation.

Upon completing the instructions, the experimenter sent the link to the online experiment. After the experiment was fully loaded in the participant’s computer, the participant was asked to contact the experimenter if (a) any problem arises during the experiment and (b) upon completing the experiment (for debriefing). Finally, the experimenter ended the video conference, and the participant began the experiment.

Following the instructions, there was a practice phase. The practice phase included 30 trials in which participants practiced (only) the visual search task and the corresponding VS cues. This phase involved ten trials of each condition: color-search, shape-search, and conjunction search. Each response in the practice phase was followed by “correct response” or “incorrect response” feedback displaying for 1,000 ms. The relatively lengthy practice phase was used to ensure participants’ familiarity with the visual search cue when performing the experiment.

Upon completing the practice phase, the experiment itself began. The experiment comprised four identically structured blocks, each block comprising 60 trials. Each trial started with a VS cue for 1,500 ms. Next, a fixation frame was presented for 1,000 ms such that its size and positioning matched the upcoming emotion-eliciting stimulus. Next, the emotion eliciting stimulus appeared for 8,000 ms. During this time, participants responded to the question (freely translated from the Hebrew original) “What sensation does the picture evoke in you?” using the designated keyboard keys: Q (left) key to report unpleasant feelings and P (right) key to report about pleasant feeling. The emotion eliciting picture stayed on screen for the entirety of the 8,000 ms regardless of the participant’s response. This procedure was deployed to prevent a strategy of responding quickly to unpleasant stimuli to terminate their presentation (Givon et al., 2020). Finally, the visual search task was displayed until the participants responded, using designated keyboard keys: Q for target absent, and P for target present.

Upon completing the experiment, participants called the experimenter for debriefing. The experimenter asked them what they thought the manipulated variables were, what they thought was the goal of the study, whether their emotional reports were authentic, and if they have any further questions regarding the experiment. None of the participants reported that s/he thought that the focus was on the emotion reports.

### Counterbalancing

We ensured that each block contained an equal number of (a) positive and negative emotional pictures, (b) an equal number of pictures belonging to each stimulus category (people/objects/landscapes/faces) within the positive and negative pictures, and (c) an equal number of stimuli belonging to each level of the visual search task (conjunction search/color search/shape search). Most crucially, we also ensured that each search task was not disproportionally preceded by a certain type of emotionally eliciting stimuli (e.g., difficult searches disproportionally preceded by negative landscapes pictures). Additionally, within each block, we ensured that no level of the search task was disproportionally preceded by a certain type of emotionally eliciting stimuli by constraining the amount of the emotionally eliciting stimuli type preceding the visual search task.

## Results

### Manipulation check

Although the analysis of the visual search data was not preregistered, we felt it is vital to demonstrate that conjunction search was more difficult than feature search. We thus analyzed mean RT and proportion of errors (PE) in the two visual search conditions. As expected, compared with feature search (*M*_RT_-color = 1,131 ms, *M*_RT_-shape = 1,449 ms, *M*_PE_-color = 0.137, *M*_PE_-shape = 0.135), conjunction search was associated with slower reaction time (*M*_RT_ = 1,832 ms); unexpectedly, it was not associated with a significantly higher error rate (*M*_PE_ = 0.139) than the feature search. We conducted a Bayesian Analysis of Variance (BANOVA) using JASP (JASP Team, 2019) with default priors to compute BF. The analysis indicated significant differences for RT (*BF* > 1,000) but accepted H0 for PE (*BF*_01_ = 6.211). Finally, to clarify the nature of the RT main effect, we conducted a two-sided Bayesian paired *t*-test on each pair of three mean RTs, indicating decisive support for a difference in each of the three pairwise comparisons (*BF*_10_ > 1,000). These results validate our key manipulation. All the remaining analyses were conducted on the emotion report data.

### Preprocessing

We executed the following pre-processing pipeline using R (R Core Team, 2014):

- **Step 1.** We excluded participants who responded counter normatively in more than 50% of trials. This resulted in the exclusion of 6 participants. In addition, we also intended to exclude participants who raised suspicions regarding the sincerity of their reported emotions (i.e., participants who indicated in the debriefing that they reported their expected rather than felt emotion). However, we did not exclude any participants on this basis, because the postexperimental debriefing revealed that all the participants indicated that they reported their authentic feelings.
- **Step 2.** We excluded ten emotional pictures (all normatively pleasant) for which the rate of normative response fell below 50%, suggesting that the norms of these pictures were inappropriate for our population, and 640 trials (10 trials per participant) involving these stimuli were excluded from further analysis (4.16% of the remaining trials).
- **Step 3.** We excluded excessively slow reactions. This was done by visually inspecting the RT density distribution, which led us to conclude that reaction time greater or equal to 6.1 s and trials with reaction times ≤200 ms deemed outliers. This resulted in the exclusion of 71 trials (0.48% of the remaining trials).
- **Step 4.** Excluding trials with extreme RT per combination of independent variables. For each combination of a participant, emotional picture, and visual search task, we excluded all trials with RTs > Z = $\left|3.5\right|$. This resulted in the removal of 63 trials (0.43% of the remaining trials).

### Analysis

Given that the model parameters are based on RT and the rate of counter normative responses, we begin with the analyses of these variables but remind the reader that the core predictions refer to model parameters and not to the raw RT and response rate.

#### Aberrant Response Rate

**Fig. 3:**
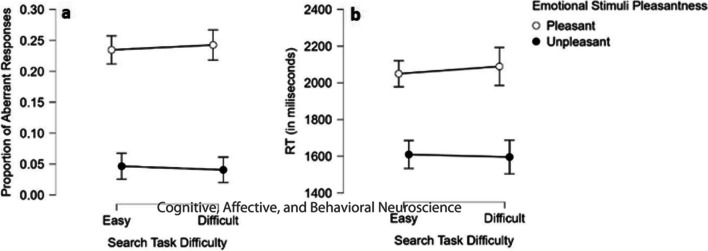
(**a**) Aberrant response rate and (**b**) reaction time as a function of the visual search task difficulty and emotional stimuli normative pleasantness. Error bars depict 95% credible intervals

Figure 3a depicts the mean proportions that were compared with a 2 (difficulty) x 2 (pleasantness) BANOVA. The analysis yielded a significant main effect for pleasantness (*BF*_10_ > 1,000), indicating a lower rate of aberrant responses for normatively negative stimuli (*M* = 0*.*044) than for normatively positive stimuli (*M* = 0.239). There was no main effect for (search) task difficulty (*M*_easy_ = 0.141, *M*_difficult_ = 0.142) with BF allowing acceptance of H0 (*BF*_01_ = 7.407). This finding means that participants were equally normative in their responses regardless of the difficulty of the upcoming search task. Finally, the interaction between emotion and search type was statistically zero with BF supporting H0 over H1 (*BF*_10_ = 4.444).

#### RT

Figure 3b depicts the means per condition. Participants were significantly quicker to respond to unpleasant (*M* = 1,602 ms) than to pleasant (*M* = 2,069 ms) stimuli (*BF*_10_ > 1,000). There was no main effect for search task difficulty (*M*_easy_ = 1,828 ms, *M*_difficult_ = 1,842 ms) with BF allowing acceptance of H0 (*BF*_01_ = 7.246). Finally, the pleasantness X difficulty interaction was not significant by NHT but had an undecided BF tending toward H1 (*BF*_10_ = 2.7).

Note that although there were not too many interesting findings related to RT/aberrant response rate, this does not imply that model estimates cannot reveal interesting patterns. One reason is that the model is not reliant on mean RT – but on the entire shape of the RT distribution.

### Hierarchical Bayesian LBA Modeling

We modeled the results using two hierarchical Bayesian LBA models: a null model and a core model. In each model, we specified the to be estimated parameters along with their prior values (Table 1). In the null model, we assumed no influence of anticipated effort and the type of emotional stimulus on the mean evidence accumulation rate or the decision boundary parameters. In contrast, the core model assumed that anticipated effort and the type of emotional stimuli have affected both the decision boundary and the mean evidence accumulation rate in both the “normative” and “aberrant” accumulators. The function of the null model was to constitute a baseline against which we compared the performance of our core model. Specifically, if the core model would outperform the null model, this would indicate a significant influence of anticipated effort on either B, mean_v, or both. We note that this is not the core test of our hypotheses given the nonspecific outcome. The test simply gives legitimacy to proceed with the (core) BANOVAs. Our modeling was conducted using the “ggdmc” R package (Lin & Strickland, 2020). This modeling involves estimating the posterior probability distributions for each parameter. This estimation is made for each participant separately but constrains this estimation with a population-level distribution. In detail, the estimation is based on drawing posterior samples taken from a hyperspace containing all possible values for all parameters.

**Table 1:** To be estimated parameters alongside their prior values for the null and core models

Parameter name	Mean prior	SD prior
**Null model**		
A	2	0.6
B	2	0.6
t0	0.3	0.1
mean_v.normative	2	0.8
mean_v.aberrant	0.5	0.8
**Core model**		
A	2	0.6
B (across all levels of pleasantness and difficulty)	2	0.6
t0	0.3	0.1
mean_v.normative (across all levels of pleasantness and difficulty)	2	0.8
mean_v.aberrant (across all levels of pleasantness and difficulty)	0.5	0.8

To sample values from the hyperspace, ggdmc uses Monte Carlo Markov Chains (MCMC). The sampling process is divided into two stages: the burn-in stage and the actual sampling stage. The burn-in stage identifies regions of the hyperspace that yield nonnegligible posterior probabilities. During the next stage, the sampling focuses on these regions in the hyperspace. We set the burn-in stage to include 1,000 samples and the actual sampling stage to include 8,000 samples. In both stages, we used 42 chains, and for the actual sampling we used thinning = 12.

### Model Adequacy and Comparison

Assessing posterior sampling adequacy was done via the Gelman-Rubin (GR) statistic (Brooks & Gelman, 1998); the statistic was separately computed for each participant in each of our models (core/null). An ideal GR score is 1.00, but scores falling below 1.10 are still acceptable. Our GR scores indicated that, indeed, our posterior sampling was adequate, because the *largest* GR score in both models was equal to 1.02. Next, we compared the performance of the core model and the null model in the following manner. First, for each participant, we calculated two Deviance Information Criterion (DIC) scores: one DIC score for the core model, and the other for the null model. Then, we summed the DIC scores for each model across participants and compared the results. The results indicated by far better performance for the core model with a (lower = better) cumulative DIC score of 39,126.97, while the cumulative DIC score for the null model was 41,066.81. This DIC discrepancy of ~1940 points is considered to be huge given that a discrepancy of even 6 points would usually be considered as indicating a real difference. The results of this stage thus give the green light to proceed to the core analyses.

#### BANOVA

**Fig. 4:**
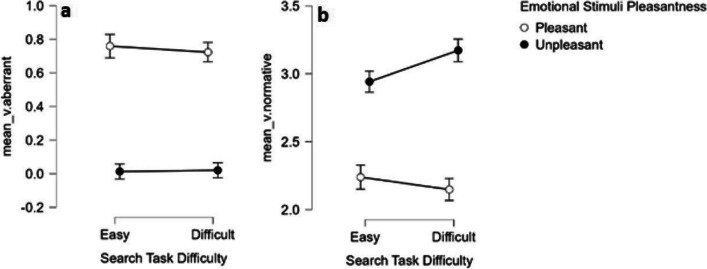
*mean_v values* as a function of condition. **a**. *mean_v.aberrant*. **b**. *mean_v. normative*. Error bars represent 95% credible interval

We conducted a separate 2x2 BANOVA on mean_v.normative and mean_v.aberrant, as estimated with the core model Fig. 4.

##### *mean_v.normative*

The analysis yielded decisive support for a main effect of stimulus pleasantness (*BF*_10_ > 1,000), such that normatively unpleasant stimuli had higher mean_v.normative (*M* = 3.05) than normatively pleasant stimuli (*M* = 2.19). More importantly, there was no support for a main effect for anticipated effort with an undecided BF (*BF*_10_ = 2.164), (*M*_easy_ = 2.59, *M*_difficult_ = 2.66). The analysis also yielded decisive support for the interaction whereby the model containing both the main effects and the interaction (H1) had greater support than the main-effect-only model (H0) (*BF*_10_ > 1,000). To clarify the nature of this interaction, we conducted two-sided Bayesian paired *t*-test for each level of search task difficulty. The *t*-test performed on the *unpleasant* stimuli showed decisive support for the difference between mean_v.difficult (*M* = 3.172) over mean_v.easy (*M* = 2.942) (*BF*_10_ > 1,000). The t-test conducted on the *pleasant* stimuli (*M*_difficult_ = 2.148, *M*_easy_ = 2.239) yielded an undecided result slightly tending towards H1 (*BF*_10_ = 2.77). These results indicate an increased evidence accumulation rate for negative stimuli in the “unpleasant” (normative) accumulator while anticipating high versus low effort. Given that mean_v is on a ratio scale where zero means zero accumulation, and with a constant unit, we can express the increase in mean_v in difficult (relative to easy) anticipated in percentage. The result shows an increase of 7.82% in the rate of negative evidence accumulation when anticipating a difficult visual search task.

##### *mean_v.aberrant*

The 2x2 BANOVA yielded only decisive support for a main effect of pleasantness (*BF*_10_ > 1,000), such that pleasant stimuli had higher mean_v.aberrant (*M*_pleasant_ = 0.742) then unpleasant stimuli (*M*_unpleasant_ = 0.017). Thus, regardless of the level of anticipated effort, evidence favoring the aberrant response accumulated faster when the target stimulus was normatively pleasant (and the aberrant response for which evidence accumulated in this case was unpleasant) than when the target stimulus was normatively unpleasant (and the aberrant response was pleasant). These results favoring evidence accumulation for unpleasant responses resemble in this respect what has been found for mean_v.normative when the normative response was “unpleasant.”

### Non-preregistered analyses

To rule out the possibility that the results reported above were confounded by the fact that the normatively pleasant and normatively unpleasant stimuli were not equidistant from neutral (5), we reran the analysis reported in the main text on a subset of stimuli with positive pictures having a valence score in the range [6, 6.5] and negative pictures having a valence score in the range [3.5, 4], thus ensuring equal distance from neutral (5). The results obtained in this analysis were almost identical to those reported above (see Supplementary Materials), besides one effect: This time we found decisive support for the effect of anticipated effort on *mean_v.normative also* for pleasant stimuli a (*BF*_10_ > 1,000). Specifically, the anticipated effort resulted in a lower mean_v (mean_v.difficult, *M* = 2.051, and mean_v.easy, *M* = 2.276), suggesting that the undecided results found in the original analysis were affected by the fact that the pleasant stimuli were too mild. Note that the new analysis has less statistical power than the original analysis given that we used much fewer trials. The fact that we found an effect in a statistically weaker analysis further strengthens the conclusions.

Finally, for the sake of completeness, we conducted another non-preregistered BANOVA on B, the boundary, that reflects speed-accuracy tradeoff. This analysis is included it in the Supplementary Materials. It indicates lower mean for unpleasant (vs. pleasant) stimuli. It also indicates that for unpleasant stimuli only, anticipated effort resulted in a higher decision boundary. In other words, the independent variables have not only influenced the rate of evidence accumulation (as predicted) but also have unpredictably influenced speed-accuracy tradeoff. This, however, does not compromise the conclusions drawn regarding the pre-registered analyses of the drift-rate.

## Discussion

The three goals of the present work were (a) to test the hypothesis that anticipated effort causes unpleasant feelings using a methodology that greatly overcomes various biases and demand characteristics; (b) further characterize “evidence” in the Feelings as Evidence Accumulation framework; and (c) to further test whether participants’ reports are about truly felt experience. To this end, we conducted a preregistered experiment. Each trial began with a VS cue indicating the difficulty level of the upcoming visual search task (either easy or difficult search). It was after the cue and during the period in which participants anticipated the visual task when the emotion evoking stimulus appeared, and participants reported whether it made them feel pleasant or unpleasant. A trial ended with the visual search task. We then fitted the LBA to the (un)pleasantness reports.

As predicted, the results revealed that anticipating greater effort has increased the rate of unpleasant evidence accumulation. Contrary to our predictions, the increase in unpleasantness only took place when unpleasantness was the normative (and expected) response, i.e., when the pictures were normatively unpleasant. There was no increase in unpleasantness when the picture was normatively pleasant. Additionally, in our first and preregistered analysis, we found that there was no effort related *decrease* in pleasantness.

After analyzing the results, we realized that there is an alternative explanation for the asymmetry of pleasantness and unpleasantness. Specifically, the normative ratings of the unpleasant stimuli (2–4) were further away from the neutral point (5) than the pleasant stimuli (5.5–6.5). One could thus argue that the pleasant stimuli were more neutral than the unpleasant stimuli. To partially deal with this issue, we reran the core analysis on half of the most pleasant stimuli and half of the least-unpleasant stimuli, such that pleasant and unpleasant were equally distant from the neutral point. The results of this analysis are reported in the Supplementary Materials and are in line with the main analysis, presented here. The exception is that this time, we found an effort related decrease in pleasantness when the pictures were normatively pleasant.

To summarize, anticipated effort has increased mean_v.normative.unpleasant and has possibly also decreased mean_v.normative.unpleasant (seen only in the non-preregistered analysis). However, it did not increase or decrease drift-rate in the aberrant responses. With respect to our goals, the results make it difficult to conclude that anticipated effort contributes to “emotional evidence” *in general*. The results nonetheless seem to “add points” to the hypothesis that participants report about their genuinely felt emotions. This conclusion hinges on the assumption that *effort related* sematic valence did not contribute to the response *to the pictures* given that the participants were instructed to report about how the pictures made them feel.

We have considered several post-hoc explanations regarding why we did not find an influence on evidence accumulation rate in the aberrant accumulator. Most of these post-hoc accounts seem not to hold. We decided to report them nonetheless to save the (convinced) readers going down these alleys.

The simplest account—the one on which we based our predictions—is that expecting a difficult visual search has led to a general negative tone (evidence favoring unpleasant feelings), but this account fails because it wrongly predicts an increase in mean.v.aberrant for normatively pleasant stimuli when expecting difficult (compared with easy) visual search. This is so, because mean.v.aberrant for normatively pleasant stimuli indicates unpleasant feelings.

Another account is that anticipating effort shifts attention to negative aspects when these are dominant, explaining why drift rate for unpleasantness increased only for normatively unpleasant stimuli. This account seems to predict that pleasantness will *not* decrease under conditions of increased anticipated effort, as we found in our registered analysis but is compatible with what we found in the non-preregistered analysis, where pleasantness was also affected by anticipated effort.

The last account, and so far, our best post-hoc guess is the intensity account. According to this account, anticipated effort does constitute a source of emotional evidence, for both lesser pleasantness and greater unpleasantness, but only when the amount of emotional evidence passes some minimal level. This account holds, because we found that anticipated effort affected (un)pleasantness only for normatively unpleasant stimuli for which the amount of unpleasant evidence is relatively intense. Similarly, we have found (in the non-preregistered analysis) that anticipated effort has reduced pleasantness for normatively pleasant stimuli. The fact that we did not find an influence on evidence for aberrant responses is explained by the fact that evidence accumulating aberrant responses is relatively weak (Berkovich & Meiran, 2023). This last account is admittedly completely post-hoc, but it is the only account we could come up with that seems to properly explain the findings. For sure, much more work is needed here.

We wish to add several reservations. The first refers to the fact that what we called “intensity” may actually be arousal. Specifically, as valence becomes more distant from the neutral point (5), the degree of arousal increases. Thus, more “intense” (un)pleasant pictures are actually also more arousing. We do not have a clear stance on this issue and leave it to the readers to choose their preferred conceptualization.

Another reservation concerns our narrow operationalization of “effort,” which actually represented cognitive load. Concluding that cognitive load is a fair representation of effort in general is a matter of leap of faith that we take but leave it to the readers to decide whether to take it.

Last, although this is somewhat obvious, we remind the reader that the conclusions were drawn based on specific stimuli (pictures), with participants who do not represent the population at large (students) while using a specific inferential scheme (LBA, RT, etc.).

## Conclusions

We found that anticipated effort has increased the rate of accumulation of unpleasant emotional evidence when the pictures were normatively unpleasant but not when they were normatively pleasant. Whether anticipated effort is a source of unpleasant emotional evidence thus depends on which interpretation is given to the findings. Nonetheless, assuming that participants reacted to the pictures as instructed, and not to the anticipated effort, the results indicate that their responses (also) reflected truly felt experienced.

### Supplementary Information

Below is the link to the electronic supplementary material.

Supplementary file1 (DOCX 88 KB)
