# Supplementary material for: Bumpy ride ahead: Anticipated effort as emotional evidence?
Source: Cogn Affect Behav Neurosci. 2024 May 14;24(4):681–93. doi: 10.3758/s13415-024-01194-9 (PMC11233335; doi:10.3758/s13415-024-01194-9)

**Supplementary Materials**

**Balancing Valence**

To rule out the possibility that the results reported in the main text were confounded by the imbalance (in terms of distance from neutral) in the emotional pictures, we re-ran the analysis reported in the main text on a subset of stimuli, as described in the main text. In this analysis, all GR fell considerably below 1.1. The BANOVAs are reported below (see Figure S1).

**mean_v.normative.** The analysis yielded decisive support for a main effect for pleasantness (*BF*_10_ > 1,000), such that unpleasant stimuli had higher mean_v.normative (*M*= 2.942) then pleasant stimuli (*M*= 2.163). The main effect for anticipated effort supported H0 (*BF*_10_ = 0.154, *BF*_01_=6.493). More interestingly, the analysis yielded a statistically significant interaction effect (*BF*_10_ > 1,000), whereby the model consisting of both the main effects and the interaction (H1) had greater support than the main-effect-only model (H0). To clarify the nature of this interaction, we conducted two-sided Bayesian paired t-test for each level of pleasantness (using the default Cauchy prior). The t-test performed on the *unpleasant* stimuli as well as the pleasant stimulim showed decisive support (*BF*_10_ > 1,000) for H1.

**mean_v.aberrant.** The 2x2 BANOVA conducted on the drift-rate of the accumulator associated with aberrant responses yielded decisive support for a main effect of pleasantness (*BF*_10_ > 1,000), such that normatively pleasant stimuli had higher mean_v.aberrant (*M*_pleasant_= 0.657) then normatively unpleasant stimuli (*M*_unpleasant_= 0.025). Thus, regardless of the level of anticipated effort, evidence favoring the aberrant response accumulated faster when the target stimulus was normatively pleasant (and the aberrant response for which evidence accumulated in this case was negative) than when the target stimulus was normatively unpleasant (and the aberrant response was positive). However, there was no main effect for anticipated effort ($BF_{10}=0.252, BF_{01}=3.896)$. Finally, there was a non-significant and undecided 2-way interaction $(BF_{10}=1.153)$.

**Figure S1**


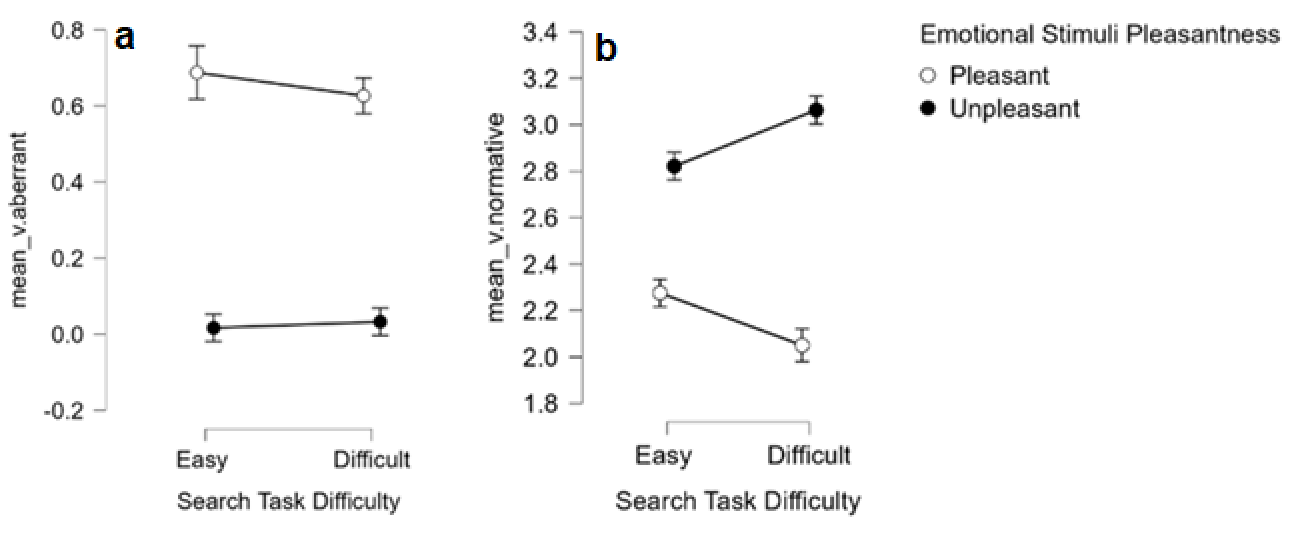


*Mean_v values of a. aberrant and b. normative responses according to difficulty and pleasantness. Error bares depict 95% credible intervals.*

**Effects on the Boundary (B)**

For completeness sake, we report the BANOVA on B. It indicated decisive support for a main effect of Valence, BF10 > 1,000, and for the superiority of main-effect+interaction model over the main-effect only model (BF=683.507). Follow up Bayesian t-tests indicate that the simple main effect of difficulty had decisive support for the unpleasant condition, BF>1,000, but was undecided and tended towards H0 for pleasant, BF_10_=0.385.


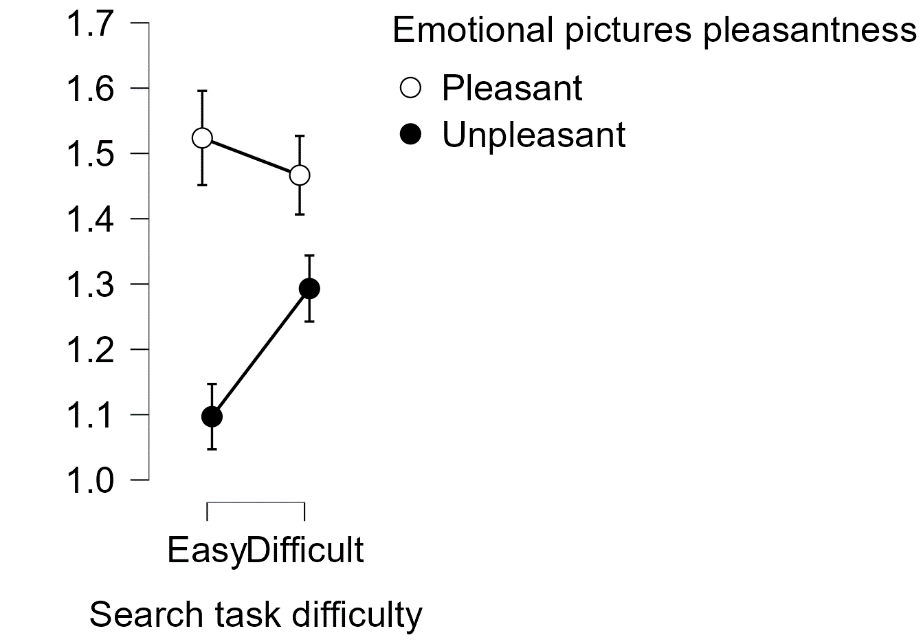

Supplement: Supplementary file 1 — Supplementary file1 (DOCX 88 KB) [file 13415_2024_1194_MOESM1_ESM.docx]
